# Detection of activities in bathrooms through deep learning and environmental data graphics images

**DOI:** 10.1016/j.heliyon.2024.e26942

**Published:** 2024-02-28

**Authors:** David Marín-García, David Bienvenido-Huertas, Juan Moyano, Carlos Rubio-Bellido, Carlos E. Rodríguez-Jiménez

**Affiliations:** a Department of Graphical Expression and Building Engineering, Higher Technical School of Building Engineering, University de Seville, 4A Reina Mercedes Avenue, Seville 41012, Spain; b Department of Building Construction, Higher Technical School of Building Engineering University of Granada, Severo Ochoa, Granada 18071, Spain; c Department of Building Construction II, Higher Technical School of Building Engineering, University de Seville, 4A Reina Mercedes Avenue, Seville 41012, Spain

**Keywords:** Activity recognition, Bathrooms, Environment, CNN image classification

## Abstract

Automatic detection activities in indoor spaces has been and is a matter of great interest. Thus, in the field of health surveillance, one of the spaces frequently studied is the bathroom of homes and specifically the behaviour of users in the said space, since certain pathologies can sometimes be deduced from it. That is why, the objective of this study is to know if it is possible to automatically classify the main activities that occur within the bathroom, using an innovative methodology with respect to the methods used to date, based on environmental parameters and the application of machine learning algorithms, thus allowing privacy to be preserved, which is a notable improvement in relation to other methods. For this, the methodology followed is based on the novel application of a pre-trained convolutional network for classifying graphs resulting from the monitoring of the environmental parameters of a bathroom. The results obtained allow us to conclude that, in addition to being able to check whether environmental data are adequate for health, it is possible to detect a high rate of true positives (around 80%) in some of the most frequent and important activities, thus facilitating its automation in a very simple and economical way.

## 1 Introduction

Home surveillance and telecare services are increasingly in demand. Thus, for example, the increase in life expectancy and the need for long-term care favour progress in this area. In this sense, automation and the so-called artificial intelligence provide various solutions to monitor activities of daily living in home settings.

On the other hand, there is increasing interest in monitoring indoor air quality, especially since the last Covid-19 pandemic, since it is proven that poor environmental quality can influence the health of residents of the said spaces.

For all these reasons, this work focusses on finding out if, with the use of basic environmental parameter sensors and a machine learning algorithm trained with the data provided by the said sensors, it is possible to detect and classify activities of interest for health in bathrooms of dwellings, in addition to monitoring the environmental quality of these spaces, which are often small in size.

Regarding the contributions of this work, it is worth highlighting that the use of environmental parameters to detect activities in bathrooms is a novel methodology and an advance in the preservation of privacy. As will be seen later, most of the methodologies presented so far are based on the collection of images or sounds, which shows possible conflicts related to said privacy. On the other hand, this study promotes the installation of environmental sensors in these spaces whose data, in addition to being used to detect the indicated activities, allow monitoring of environmental quality, which is not possible with the methodologies detected to date. Furthermore, the use of machine learning algorithms greatly facilitates automatic detection and subsequent treatment. For all these reasons, the proposal represents an increase in data and at the same time a reduction in the cost of equipment.

As evidence of what is indicated, the background is first analysed as the basis for the technological and experimental development carried out.

### 1.1 Background

Wang, J., Chen, Y., Hao, S., Peng, X., & Hu, L., have recently stated in their paper “Deep learning for sensor-based activity recognition: A survey” [1], that Human Activity Recognition (HAR) [2] plays an important role in the daily lives of many people.

Currently, the main technological methods for recognising human activity (HAR) are based on images and/or sensors [[3], [4], [5]].

On the other hand, these technological methods for HAR have been applied frequently in homes [6], their bathrooms being precisely one of the spaces whose activities have been monitored on several occasions [[7], [8], [9], [10], [11]].

The subject of bathrooms and their use can be treated from different points of view, one of the most interesting being related to the automatic recognition and classification of the main activities that take place inside them, especially for monitoring people's health and telecare [12,13].

Therefore, the detection and monitoring of these activities has been the object of study using technologies such as sound capture [7,8], images or infrared [9], wearable accelerometer [10], wearable sensors [11], among others. In these cases, although quite interesting results have been achieved, there are limitations.

In Table 1 you can see a summary of the state of the art of more recent works.

**Table 1:** Summarised state of the art of most recent works related to activity detection, especially in bathrooms.

Id	Authors	Year	Tittle	Publication	Topic
**1**	M.H. Arshad, M. Bilal, A. Gani	2022	Human activity recognition: Review, taxonomy and open challenges [5]	Sensors	A
**2**	D. Bouchabou, S.M. Nguyen, C. Lohr, B. Leduc, I. Kanellos	2021	A survey of human activity recognition in smart homes based on IoT sensors algorithms: Taxonomies, challenges, and opportunities with deep learning [6]	Sensors	A
**3**	B. Fu, N. Damer, F. Kirchbuchner, A. Kuijper	2020	Sensing technology for human activity recognition: A comprehensive survey [3]	IEEE Access	A
**4**	Y. Zhang, I. D'haeseleer, J. Coelho, V. Vanden Abeele, B. Vanrumste	2021	Recognition of Bathroom Activities in Older Adults Using Wearable Sensors: A Systematic Review and Recommendations [11]	Sensors	B
**5**	Y. Zhang, J. Wullems, I. Dihaeseleer, V. Vanden Abeele, B. Vanrumste	2020	Bathroom activity monitoring for older adults via wearable device [10]	IEEE International Conference on Healthcare Informatics	B
**6**	K. Chapron, P. Lapointe, K. Bouchard, S. Gaboury		Highly Accurate Bathroom Activity Recognition Using Infrared Proximity Sensors [9]	IEEE Journal of Biomedical and Health Informatics	B
**7**	J. Chen, A.H. Kam, J. Zhang, N. Liu, L. Shue	2005	Bathroom Activity Monitoring Based on Sound [8]	Pervasive Computing: Third International Conference, PERVASIVE 2005	B

A) Technology for the recognition of human activity in general.

B) Technology for the recognition of human activity in bathrooms.

Thus, although privacy is tried to preserve [14], it is generally a problematic issue, especially in the case of capturing images and sounds. Even when the proposed system includes devices that the user must always carry [10,11], these limitations occur because they require consent and commitment to use.

For all these reasons, it is necessary to continue to advance in the development of these automatic detection systems, even more so when no studies have been detected on the relationship between environmental parameters and user performance in residential bathrooms in terms of automatic detection.

Therefore, this study focusses on recording the most common performances inside the bathroom, their duration, and the environmental parameter values (including CO_2_ levels, temperature, relative humidity, dew point, and wet bulb) to find out if it is possible to automatically identify these performances.

For this automatic classification, some authors mentioned have applied automatic learning algorithms with the collected data [10,11], but none have carried out a classification using environmental parameters and the images of the graphs generated by the time series corresponding to the values of said parameters, applying convolutional neural networks pretrained designed for image classification.

Regarding deep learning with convolutional neural networks, it has undergone rapid evolution and improvement in the last decades with various architectures and numerous applications have emerged [[15], [16], [17]], such as voice recognition [18,19], classification of handwritten characters [20,21], facial recognition [22], o activities and behaviour [23], translation and interpretation of texts [24], computer vision and defect detection [25,26], classification of images with one or multiple labels [[27], [28], [29], [30]], and in various fields of study such as medical [31], or satellite imagery [32], among others [33].

On the other hand, the use of the technique of taking advantage of the pre-training of a convolutional neural network (Transfer Learning) to reduce training times by trying to maintain or even improve the accuracy of image classification has been frequently applied [34].

This study focusses on experimenting with the application of a pre-trained neural network to find out if it is possible with it to classify representations of time series of values of the environmental parameter that allows the classification of certain activities of interest in bathrooms of the dwellings.

Thus, the novelty presented by this research is the use of the image classification methodology and not that of key values. That is, in this study, the environmental data are expressed through time series and to carry out the task of classifying the said series they are processed as images, something like spectrograms, and with them a convolutional model is trained.

There are several examples of the application of this technique in other areas, such as recognising handwritten numbers, handwritten signatures, drawings, etc. [[35], [36], [37], [38], [39], [40]], but none have been applied to this field of environmental parameters in residential bathrooms.

It is true that due to its simplicity can work well to train a convolutional model, but instead of using 2D convolutions (for images), using 1D convolutions (for sequences) [41].

However, this last methodology, even though it is faster to train, needs the same times and circumstances of all the cases; otherwise, it is not as precise when it comes to detecting patterns, which is why in this research it is decided to apply the first of the aforementioned methodologies in such a way that with the graphs generated and transformed into digital images, related to relative humidity, as will be seen in the results, the dataset is made by organising it into conveniently labelled folders, allocating images for training (approximately 80%) and for validation (approximately 20%).

In short, it is significantly innovative that a deep learning algorithm is used for image classification so that, once trained, it can analyse the images of environmental data graphs from the interior of a bathroom and deduce from them the activities that occur in its interior, being able to simultaneously control the environmental health and certain aspects of the users' health, in both cases and with the appropriate programming, automatically or semi-automatically.

### 1.2 Reason for their new image classification methodology

This innovation is highly competitive with respect to other methodologies studied since, unlike the model formulated in this work, none of them allows, at the same time, the preservation of 100% of privacy, a minimum cost of use (simple sensor of CO_2_, relative humidity and temperature) and monitoring telematics, as well as the possibility of training the algorithm to customise it for each specific space and user or users.

Therefore, this study differs from other recent in that i) the user does not need to carry any device; ii) the data that is recorded gives us an idea of the quality of the air; iii) it guarantees total privacy (is not the case of images or sounds); vi) it is the result of a novel image classification procedure using convolutional neural networks and images of time series graphs. Despite all this, it should be noted that the studies already carried out have been beneficial for the development of this work in the sense of providing the basis for understanding that it is possible to distinguish activities in bathrooms by recording time series, as is the case of the recording of sounds, and how it is also possible to apply machine learning algorithms to the results, which needs to be perfected in the aspects indicated above.

### 1.3 General properties of deep learning

In the case study, “deep learning" is a very powerful tool for analysing data such as time series, allowing us to understand and work with unstructured information effectively, such as images or graphs with subtle elements, recognising patterns and complex relationships or connections between the data, and even through strategies such as transfer learning, a model can be obtained that can take advantage of the knowledge acquired from a task to apply it in another, as is the case of this work.

However, they are not all advantages, since these algorithms sometimes present an added difficulty in constructing the appropriate data set with impartiality, sufficient size, varied, and without errors. Despite this, it is undeniable that the advantages outweigh the disadvantages in most of the fields in which it is applied, as is the case of this study.

## 2 Materials and methods

### 2.1 Experiment bathroom

Given that bathrooms can have variable characteristics, especially in terms of ventilation [42] and dimensions, this study is a pilot experiment, that is, it is about monitoring a bathroom with specific characteristics, with the sole purpose of finding out if there is reasonable evidence to deduce that the methodology used is applicable to other bathrooms with different characteristics.

On the other hand, the chosen bathroom does not have a window or an extractor fan [43], and although the door opens and closes when entering and leaving, during the time of use of the bathroom, the door is closed, as it is considered that with an open window or door, the parameters could be too variables and unpredictable, so the results of this study are limited to this type of bathroom.

Regarding the bathroom extractor, it is likely that the methodology of this study is applicable to bathrooms with extractor fans if these are automatically on and have a stable flow rate.

**Fig. 1:**
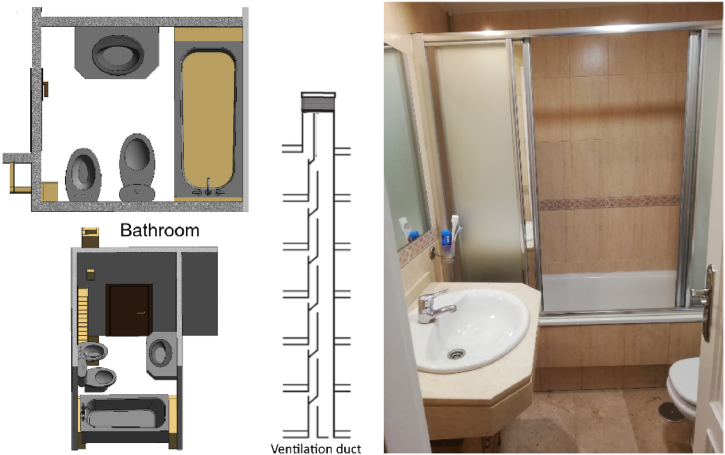
Characteristics of the bathroom.

Taking these conditions into account, the chosen bathroom dimensions are 1.83 × 2.03 × 2.33 m (8.68 m^3^ of indoor volume); there is a front door of 0.62 × 2.03 m, with a distance between the floor and its low-end section of 5 mm, and a ventilation duct, shared with other dwellings, connected with a vent on the roof of the building and with the characteristics specified in Fig. 1.

The dimensions of the ventilation vent on the wall of the bathroom are 10 × 14 cm.

Spatial characteristics are reliably represented by a three-dimensional model designed with Autodesk Revit 2019® [44].

The experimentation took place from October to December 2020 and from January to February 2021, in the south of Spain.

On the other hand, of all the data records made, 613 were chosen to make the data set as balanced as possible between activities to ensure the reliability of the results of the training of the convolutional network.

### 2.2 Equipment

To measure indoor and outdoor temperature, relative humidity, and CO_2_ concentrations, an indoor air quality metre with a nondispersive infrared sensor was used: TSI IAQ-CALC™ Model 7525 (TSI Incorporated, Shoreview, MN, EUA), whose technical characteristics are shown in Table 2.

**Table 2:** Specifications of the indoor air quality metre used: IAQ-CALC™ 7525.

CO_2_		Temperature	Relative Humidity	Outdoor Air Percentage
Sensor Type	Dual-wavelength NDIR (non-dispersive infrared)	Thermistor	Thin-film capacitive	
Range	Range 0–5.000 ppm	0–60 °C	5%–95% RH	0–100%
Accuracy	±3.0% of reading or ±50 ppm, whichever is greater	±0.5 °C	±3.0% RH	
Resolution	1 ppm	0.1 °C	0.1% RH	0.1%
Response	20 s	30 s (90% of final value, air velocity at 2 m/s [400 ft/min])	20 s (for 63% of final value)	

All measurements were performed according to the following specifications: i) placing the sensor in a place with less ventilation and, therefore, far from the door or airflows so that the measurement is as restrictive and representative as possible of the air quality being breathing in the room; ii) keeping a certain distance between individuals and the place where the metre is placed because breathing could affect the records; and finally, performing continuous measurements (the metre records measurements every 20 s).

**Fig. 2:**
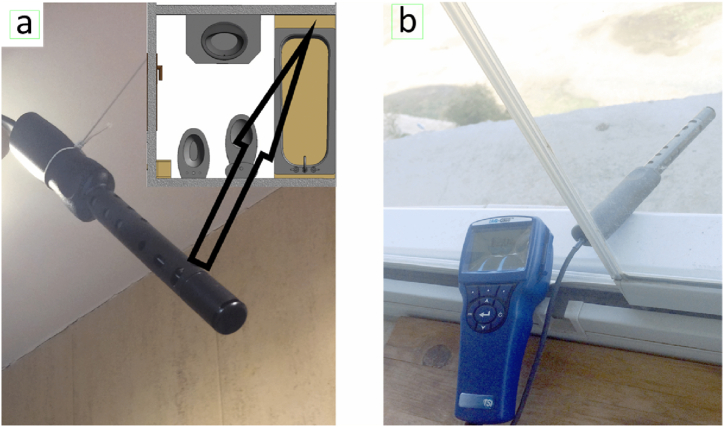
(a) Position of the measurement probe inside the bathroom and (b) outside the dwelling.

Furthermore, environmental data outside were collected before, simultaneously, and after experimentation (Fig. 2).

To verify the ventilation and the presence of the user, a Hot Wire Thermo-anemometer of Extech Instruments, Model SDL350, and an HC-SR501 presence sensor on the ceiling of the bathroom connected to Arduino were also used (Table 3).

**Table 3:** Specifications of the Hot Wire Thermo-anemometer of Extech Instruments, Model SDL350 and HC-SR501 presence sensor.

Hot Wire Thermo-anemometer of Extech Instruments, Model SDL350			
Specifications	Range	Resolution	Basic Accuracy
Air Velocity m/s	0.2–25 m/s	0.01 m/s	±5%rdg
ft/min	40–3940 ft/min	1 ft/min	±5%rdg
MPH	0.5 to 45MPH	0.01MPH	±5%rdg
knots	1 to 31knots	0.01knots	±5%rdg
km/h	0.7–72 km/h	0.01 km/h	±5%rdg
CFM (meter^3^/min)	0 to 54,000 CFM	0.001 to 100 CFM	
CMM (meter^3^/min)	0 to 54,000 CMM	0.001 to 1 CMM	
Temperature	0–50 °C	0.1°	±1.5 °F (±0.8 °C)
Type K Temperature	−100 to 1300 °C	0.1°	±(0.4% + 1 °C)
Type J Temperature	−100 to 1200 °C	0.1°	±(0.4% + 1 °C)
**HC-SR501 presence sensor**			
**Specifications**	**Range**		
Detection angle	120°		
Configurable delay	Between 5 and 300 seg		
Maximum distance	7 m		
Operating mode	Continuous-repeat		
Sensitivity adjustment	Yes		
Timer setting	3 seg. to 5 min		

### 2.3 Bathroom ventilation calculation

After performing certain tests with simulation software such as CONTAM® [45], it was decided that, since CO_2_ measurement equipment was available, ventilation could experimentally be calculated using the steady-state method of CO_2._ [[46], [47], [48]].

With the bathroom occupied, the measurement by means of a CO_2_ sensor allows knowing the approximate ventilation. If enough time elapses, steady-state CO_2_ concentration could be obtained and the renewal rate (ACH) can be deduced.

Equation (1) allows to calculate the steady-state CO_2_.

(1) $$\text{Steady}\;\text{state}\;\text{CO}2\;\left(\text{SsCO}2\right).=\left({Co}_{2\;gr}+\left(Af*\;Caf*\;0.000001\right)\right)/\left(Af*0.000001\right)$$

where Co_2_ gr = CO_2_ generation rate. This is calculated by multiplying the number of occupants by their CO_2_ exhalation rate. The rate should oscillate between 0.24636 and 0.36812 depending on age and activity (breathing, talking, etc.). For example, for an adult CO_2_ gr = 0.36812 L/min. Af = Air flow= (((vol*ACH)/60 m^3^/minute) *1000 lpm-litres/min). In the case of study vol = 8.68m3. Thus, ACH=(Af m^3^/minute * 60)/vol m^3^. Caf = Outdoor background concentration in ppm. It is around 400 ppm. The average of the measurements of the CO_2_ levels recorded outside was 393 ppm.

Thus, as in the case of the study, SsCO_2_ is known experimentally and can also find out “Af", and with it the air changes per hour (ACH).

Equation (2) allows to calculate the Af in lpm.

(2) Af=0,36812/((SsCO2*0.000001)−(393*0.000001))

ACH was 0.75 for Af = 108–109 lpm in the most unfavourable case with a SsCO_2_ around 3500 ppm.

Furthermore, to confirm the calculations, the corresponding checks were also carried out with the Extech Instruments Model SDL350 hot wire thermoanemometer.

### 2.4 Experiment

Basically, it is about monitoring the environmental parameters of the chosen bathroom.

Subsequently, a dataset is created with the data collected in the form of graphic images that represent different time series of the values of said parameters, to subsequently select those that are most appropriate (the patterns that are more evident visually) to train a supervised learning algorithm (image classifier using a pre-trained convolutional network) analysing the results in order to find out if the precision is enough to detect and classify the different activities that occur in the bathroom. In this sense, it should be noted that although the methodology of using only key values from which patterns can be deduced from the time series could have been followed, it was decided to use artificial vision as an innovative technique for classifying the images of the temporary graphs created from the records of environmental parameters.

In the first phase, the activities to be detected during the performances are selected. Performances are understood as the set of activities carried out from the moment they enter the bathroom until they leave it. Within each action, the specific activities that it wanted to detect, due to their clear impact on people's health, principally are: only defecating (0), only urinating (1), only washing hands (2) defecate and shower (3) urinate and shower (4) only shower (5) only brush teeth (6). In response to Barker and Jones's [49] recent comment on the possible spread of infection by aerosol contamination after flushing a household toilet with the lid open, a device invented by one of the research authors is installed in the toilet, which does not allow the toilet to flush without closing the lid.

To clarify in more detail what the activities in the bathroom were like, it must be indicated that defecating (0) and urinating (1) consisted of initially opening and closing the door of the bathroom, proceeding to defecate, wipe with toilet paper or urinate, if applicable, handwashing hands to then dry them and proceed to leave the room, leaving the door closed; only hand washing (2) consisted of entering, washing and leaving in the same way as in the previous case; defecate and shower (3) and urinate and shower (4) the same as in case (0) but including showering and drying; only shower (5) too the same as the previous one but without defecating or urinating; simply brush your teeth (6) the same process as washing your hands.

The selected performances always started by entering the bathroom and ended by leaving it, although data was recorded before and after these moments.

Moreover, outside environmental data were collected before, simultaneously, and after experimentation, as well as data to know the steady state CO_2_ equilibrium concentration.

The study was complemented by post hoc manual verifications to check the results, detect incidents, and analyse and correct them, although none distorted the measurement results.

### 2.5 Data set organization and classification algorithm application

Before the final data set of images is made, the graphs are visually analysed to find out which of the parameters is of greatest interest for the purposes of the intended classification. In this case, as will be seen in the results section, the relative humidity graphs are those that present characteristics that make it possible to better differentiate a large part of the activities that are intended to be detected. For this reason, these data will be used mainly to train and validate the proposed classification model. In this regard, it could be questionable why relative humidity, which can depend on temperature, is used instead of moisture content or partial vapour pressure. The answer lies in the results of the measurements, which showed that the dry bulb remained practically constant in all the activities analysed (except when users shower). Furthermore, the range of temperature oscillation during activities is minimal (except when users shower). Therefore, the exclusive use of relative humidity implies representative results.

With the graphs generated and transformed into digital images, the data set is organised into conveniently labelled folders, allowing 672 images for training (approximately 80%) and 161 for validation (approximately 20%).

Since these are time series chart images, some data augmentation techniques are employed, such as rotating, flipping, mirroring, etc. will not be applied. However, resizing techniques will be applied so that the images are uniform (all images will be resized to 224 × 224) and batch random. Regarding the validation data set, the images will not be transformed or mixed.

Of the different technological tools currently available for use in neural network projects, this research uses the Keras neural network library [50], supported by TensorFlow [51]. Keras provides several pre-trained neural networks so that you only need to import and use them.

Normally, the pre-trained Keras network is chosen that, through the comparative study, gives the best results or that appears in the ranking for the ImageNet [52] validation data set. (precision top-1 y top-5) [53]. For the case study, it is understood that if the results allow conclusions of interest to be drawn with a pre-trained network with a somewhat lower top than the rest of those proposed by Keras, with the rest of the architectures the results will be similar or better, so that there is no need for all the architectures offered by keras, at least considering the objective pursued. Following this reasoning, the pre-trained VGG16 network has been chosen, which, having been already proven for several years, is one of the simplest to apply and, although it has been surpassed in training with ImageNet [54], by others (ResNet, Origen, InceptionResNet, MobileNet, DenseNet, NASNet, EfficientNet), it can be a good indicator of what the rest of the architectures will present.

Regarding the necessary pre-processing work, it basically consists of transforming the data of the images of the graphics with a unified format, making use of the instructions provided by Keras to transform the form of the input data.

To do this, the corresponding notebook is created and the Keras library is loaded, after which the data loading process is executed, separating the various parts of this dataset: (training set and validation set). Each of these data sets (images) have their respective labels.

On the other hand, regarding the Transfer Learning process, it begins with feature extraction, which consists of using representations learnt by another network to extract interesting features from the new data set. After this extraction, the new representation given by these features is used to feed a new classifier.

The CNNs used for image classification consist of two well-differentiated parts: i) they begin with a series of alternating layers of convolution and pooling (what is called the convolutional base) and end with a classifier of dense layers.

For Transfer Learning, the convolutional base of the pre-trained network is used, containing information about generic concepts, which seem useful for all kinds of computer vision problems, so only the last part of the network will be trained.

It must be remembered that the level of use of the convolutional basis depends on the depth of the model layers. In the case of time-series studies, the first layers are usually the ones of greatest interest since they extract maps of very generic and local characteristics (edges, colours, textures, etc.) although the following layers can also contribute; they are more specific to extract characteristics of more specific elements (objects, organs, etc.).

Therefore, for the case study, the new dataset is very different from the dataset with which the network was initially trained, and therefore only the first layers are the most appropriate to use in Transfer Learning. With this premise and once the VGG16 pre-trained network was loaded, the compilation and training of the data set of the images of the time series were carried out by freezing the convolutional base, that is, the weights of its layers.

Regarding the steps by season and batch size, after the appropriate tests are presented in the following results section.

**Fig. 3:**
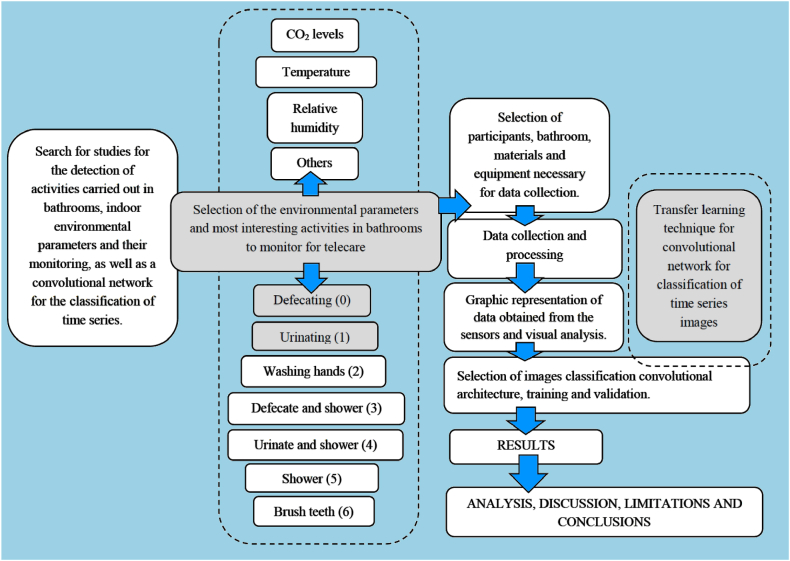
Conceptual flowchart of the methodology. Steps followed in the research.

Fig. 3 shows the summary of the steps followed for the development of the chosen methodology.

## 3 Results

### 3.1 Graphic representation of the data obtained and visual analysis

**Fig. 4:**
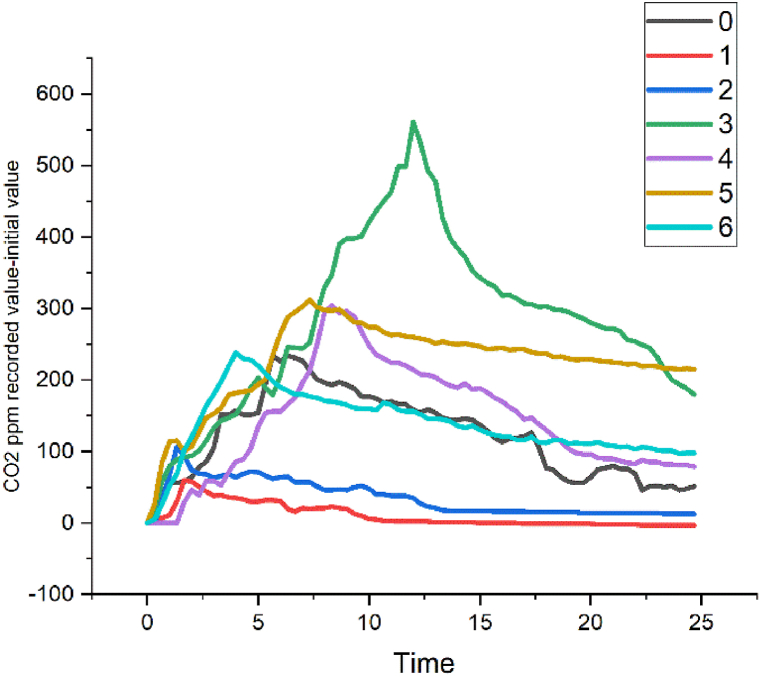
Examples of some results in absolute values (recorded value-initial value) of the CO_2_ level of the experimental tests. Activities 0 to 6 are presented.

Fig. 4 shows graphic examples of CO_2_ levels resulting from the experimental tests carried out for each of the activities. These results are reflected with absolute values (recorded value-initial value) plotted with equal time intervals on the x-axis and absolute values of CO_2_ on the y-axis.

From the visual analysis of the graphs, it can be clearly reduced that, for the purpose of detecting the different activities, the graphs of the CO_2_ time series are not adequate, since, from the moment the user enters the bathroom until the maximum level is reached or this user leaves it, the graph plot is similar, only shorter or longer, simply depending on the length of time the user stays inside the bathroom [48].

However, from the observation of the presence detector and the CO_2_ levels data, it can be deduced that based on the data on the moment in which the CO_2_ levels begin to rise and the moment in which the maximum level of concentration of said gas is produced, the duration of the complete performance could be calculated. The precision of this deduction is high, since there is a lag at the beginning of the performance that is compensated for by the lag at the end.

So, with the monitoring of CO_2_ levels, of which it should be noted that no levels considered risky in relation to exposure time were recorded [55], it is possible to obtain data on the presence of a user, bathroom ventilation, and duration of performance, in the latter case with a margin of error between 1 and 5% of the time elapsed from the time that said CO_2_ begins to increase until the levels begin to drop.

**Fig. 5:**
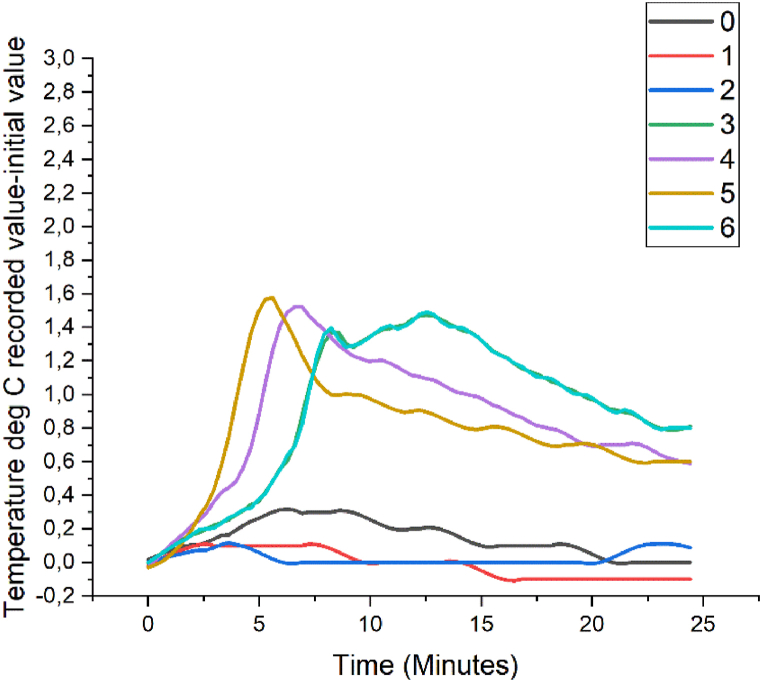
Examples of some results of the experimental tests temperature. Activities 0 to 6 are presented. The graphs reflected absolute (recorded value-initial value).

Regarding temperature, Fig. 5 shows graphic examples of temperature resulting from the experimental tests carried out for each of the activities. As in the case of CO_2_, these results are reflected with absolute values (recorded value-initial value) plotted with equal time intervals on the x-axis and absolute temperature values on the y-axis.

From the visual observation of the temperature graphs it can be deduced that the performances in which the user showers have a well-differentiated curve with respect to the rest of the actions, which is sometimes ascending (shower with hot water) and sometimes descending (shower with cold water), so it could be of interest for detecting when users take a shower. As will be seen in the following, relative humidity levels also allow for this classification.

**Fig. 6:**
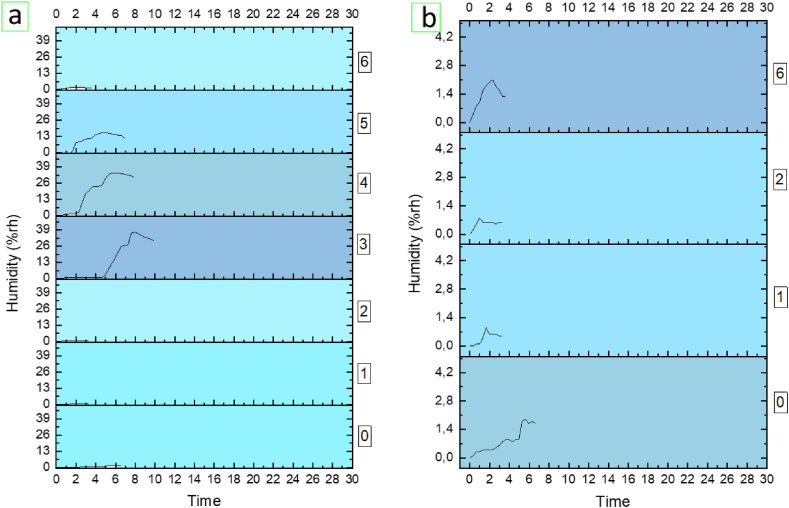
Example of some results relative humidity (absolute values: rise of relative humidity above initial value). To facilitate visual analysis, the graph on the left (a) offers the representation of results for activities 0 to 6. The graph on the right (b) offers the enlarged representation of activities 0, 1, 2 and 6.

Finally, in Fig. 6 you can see an example of the graphs of the relative humidity levels resulting from the experimental tests carried out for each of the activities if we only consider the data from when the performance begins until shortly after reaching the maximum.

These results are reflected with absolute values (recorded value-initial value) plotted with equal time intervals on the x-axis and absolute values of relative humidity on the y-axis. Since the interval between the minimum and maximum value of relative humidity when activities 0, 1, 2 and 6 occur is much smaller than that of the rest of the activities, these graphs have been enlarged as can be seen in the example of Fig. 6.

From the observation of these graphs, it can be deduced that humidity is the most important parameter on which to base oneself to distinguish the different activities 0 to 6 in the performances.

Thus, we can see clearly that there are certain patterns that identify each activity in the performance. That is why precisely these graphs are used for the training of the convolutional neural network, which is intended to result in a trained algorithm to automatically classify these activities.

### 3.2 Data set, application of the algorithm and results of the training and validation

Taking into account all of the above, once all performance and activities 0 to 6 have been monitored, a data set is created.

Due to the fact that the goal is to find out if it is possible to detect and classify the performances that occur in the bathroom by user's moments after leaving the bathroom, this study focused on the data produced during the use of the bathroom and shortly thereafter.

**Fig. 7:**
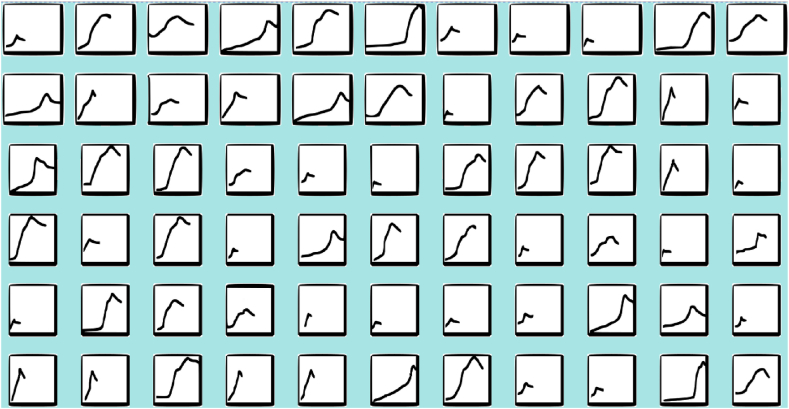
Example of part of the 833 plots images of recorded relative humidity data used to build the dataset.

As mentioned above, it can be deduced from the graphs that the key parameter is relative humidity, so with the graphic data from its monitoring, during each performance, the chosen classification convolutional architecture has been trained using the methodology described above, using 833 graphs (Fig. 7) of relative humidity corresponding to the performance with activities 0 to 6 classes.

Details of the data numbers used in this work are shown in Table 4.

**Table 4:** Details of the numbers of data used in this work.

	For training	For validation	Total
On average in every performance (Tolerance + -5)	96	23	119
Total number	672	161	833
%	80	20	100

**Fig. 8:**
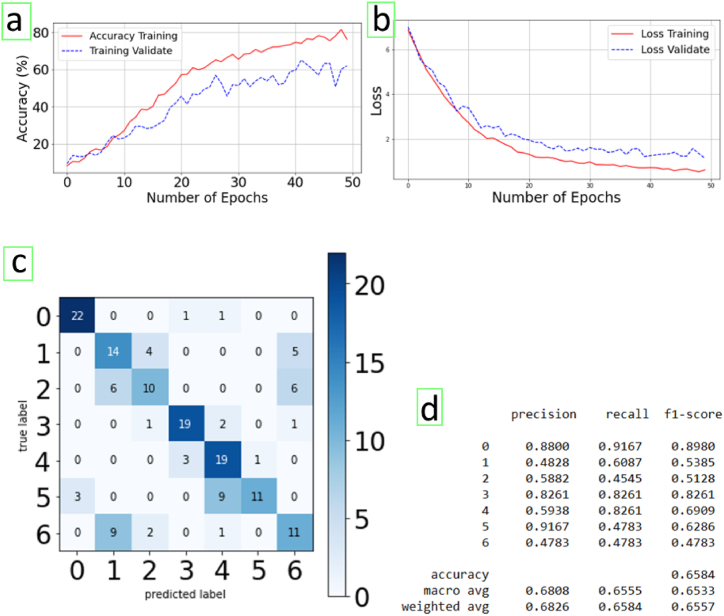
(a) Accuracy and (b) loss training-validate in each epoch. (c) Confusion matrix of activities: Only defecate (0), only urinate (1), only wash hands (2) defecate and shower (3) urinate and shower (4) only shower (5) only brush teeth (6). (d) Metrics numerical results.

Under these conditions, Fig. 8 shows the results after training and validation using the transfer learning technique with VGG16 (epochs = 50; batch size = 32), using the 7 mentioned classes in folders containing the graphic images previously adjusted to a size of 224 × 224 (Table 5). The maximum of 50 epochs was due to the availability of resources and because it was detected that the results did not improve much when the epochs were increased.

**Table 5:** Characteristics and hyperparameters used for the model and its training and validation.

Imports made from Keras	Sequential, Model Conv2D, MaxPool2D, Dense, Flatten, Dropout, BatchNormalization, Input Optimizers Adam TensorBoard, ModelCheckpoint np_utils image Applications.imagenet_utils import preprocess_input, decode_predictions VGG16 ImageDataGenerator
**Othes imports**	Os Numpy as np from sklearn.utils import shuffle from sklearn.model_selection import train_test_split cv2 Matplotlib.pyplot as plt %matplotlib inline
**Parameters**	width_shape = 224 height_shape = 224 num_classes = 7 epochs = 50 batch_size = 32
**Dense layers for use into pre-trained architecture VGG16 with imagenet**	x = Flatten (name = 'flatten') (last_layer) x = Dense (128, activation = 'relu', name = 'fc1′) (x) x = Dense (128, activation = 'relu', name = 'fc2′) (x) out = Dense (num_classes, activation = 'softmax', name = 'output') (x) custom_model = Model (image_input, out) custom_model.summary (**) # Because it is a pre-trained network to train with our graphs, all layers are frozen except the last dense ones. for layer in custom_model.layers [:-3]: layer.trainable = False custom_model.summary (**) custom_model.compile (loss = 'categorical_crossentropy',optimizer = 'adadelta',metrics = ['accuracy'])

In Fig. 8 the precision of the training in some periods reaches around 80% for the accuracy training and 70% for the training validation, with the loss training curve close to that of the loss validation. This means that with a low-density data set like the one used, interesting results are achieved.

Regarding each of the activities and their classification, in Fig. 8 the behaviour of each of them can be observed in the corresponding confusion matrix.

In the confusion matrix (Fig. 8) it is observed that the recall is above 80% in the detection of activities 0 (only defecating), 3 (defecating + shower), and 4 (urinate + shower). At the other extreme, a very low recall has been produced in the performances in which activities 5 (only shower) and 6 (only brush teeth) are carried out. This is because activity 5 is often confused with 4 (urinate + shower) and sometimes with 0 (defecate). Lastly, activity 2 (washing hands) is often confused with activity 6 (brushing teeth) and activity 2 in turn is frequently confused with activity 1 (urinate).

### 3.3 Analysis of training results and validation of the algorithm

The most remarkable of the results is that, with an accuracy of between 70 and 80%, it is possible to automatically distinguish the main activities (defecating and shower). However, the accuracy is low in the case of the activity of urinating, confusing it with hand washing and brushing teeth, due, as is logical, to the fact that these situations can generate similar values of the environmental parameters under equal circumstances.

Regarding the shower, it is clearly seen that when this activity is carried out alone or even combined with others, the algorithm distinguishes it with high precision due to the marked rise in relative humidity, although sometimes the activity of just showering is confused with urinating and showering.

However, the detection of this showering activity is clearly reinforced by a sudden change in ambient temperature, which makes it easier to classify if combined with these data.

It is true that there are activities in which the algorithm has low precision and is often confused with others, such as urinating and washing hands, as the execution times and environmental parameters are usually similar. But precisely the execution time could also improve the classification of some activities, such as those of medium or long duration (defecating, showering) from those of short duration (washing hands, urinating). In this sense, it must be remembered that the trend changes in the CO_2_ level graph are adequate for an approximate calculation of the duration of the performance.

## 4 Discusión and limitations

First, it should be said that a dataset that could be considered relatively low density has been used. However, it is sufficient to meet the goal of verifying the viability of the methodology, without prejudice to the intention to continue collecting data in the future to improve the training results. It would also be interesting to collect data from various types of bathroom to make a collection of algorithms trained according to each type.

On the other hand, the percentage of success obtained for each activity in studies carried out by other authors with sound sensors [7,8], images [9], and other procedures [10,11], is around 90% and sometimes even 100%. This could lead one to think that the study carried out does not offer advantages compared to the previous ones.

However, as has already been mentioned, in addition to offering total privacy and environmental data of significant interest that these methodologies do not offer, the goal is to verify whether the installation of environmental sensors can also serve to monitor, at least in a basic and indicative way, the most important activities of bathroom users in relation to telecare, because it should be remembered that this study is a limited pilot experiment that does not seek to find and train a universal algorithm or all the necessary algorithms that serve to classify certain activities that take place in all types of domestic bathrooms. What is really intended is to check whether, with a set of time series of environmental data, especially those related to the relative humidity of a bathroom with certain characteristics and whose limitations in their relationship with temperature have already been exposed and justified above, it is possible to carry out an approximate automatic classification of these activities.

In other words, it is confirmation of how it could be carried out, since if the ventilation and the characteristics of the bathroom vary, the data would have to be taken again and the algorithm would be retrained, although once the results of this study are known, these tasks could be carried out in a more focused and simple way, even being able to assemble a device already pre-configured to capture the data, train the algorithm, and then apply it automatically telematically, to know if the environmental parameters are adequate for the health of users [55,56], and when and how they use the bathroom.

## 5 Conclusions

The main conclusion is that monitored environmental parameter data, in addition to allowing the monitoring of air quality in the bathroom, can help distinguish activities of interest for telecare. However, not all of these activities can be detected with the same precision.

Thus, with the results of the classification, fundamentally from the graphs of the time series related to relative humidity, with an accuracy greater than 70% usually used by the referenced authors for this type of work (around 80%), although not as high as in studies carried out with other types of sound sensors, images, etc., it can detect when a user defecates or showers, two very important activities to deduce the health status of the user. On the other hand, the precision increases if the temperature and duration of the action (short, medium, or long duration activities) are also taken into account. In this sense, with data on CO_2_ levels, it is possible to obtain the presence of a user, the ventilation of the bathroom, and the duration of performance, in the latter case with a margin of error between 1 and 5% of the time elapsed from the time CO_2_ begins to increase until levels begin to drop.

Therefore, with the installation in a bathroom of CO_2_, temperature, and, above all, relative humidity sensors, which are currently very cheap, they could be registered and sent the data to any computer with a connection to the internet, with the algorithm installed, and track, with great precision, at least some of the main activities carried out in bathrooms like defecates or showers, maintaining the total privacy of the users.

All of this, it is sufficient to claim that the goal of verifying the viability of the methodology has been fulfilled, without prejudice to the intention to continue collecting data in the future to improve the results of the training.

## Ethics

Not applicable.

## Funding

The author(s) disclosed receipt of the following financial support for the research, authorship, and/or publication of this article: VI Own Research and Transfer Plan of the University of xxxx.

## Data availability statement

The data used are confidential and in principle not available, but exceptionally they may be made available upon reasonable request.

## CRediT authorship contribution statement

**David Marín-García:** Writing – review & editing, Writing – original draft, Visualization, Validation, Supervision, Software, Resources, Project administration, Methodology, Investigation, Funding acquisition, Formal analysis, Data curation, Conceptualization. **David Bienvenido-Huertas:** Writing – review & editing, Writing – original draft, Visualization, Validation, Supervision, Software, Resources, Project administration, Methodology, Investigation, Funding acquisition, Formal analysis, Data curation, Conceptualization. **Juan Moyano:** Writing – review & editing, Writing – original draft, Visualization, Validation, Supervision, Software, Resources, Project administration, Methodology, Investigation, Funding acquisition, Formal analysis, Data curation, Conceptualization. **Carlos Rubio-Bellido:** Writing – review & editing, Writing – original draft, Visualization, Validation, Supervision, Software, Resources, Project administration, Methodology, Investigation, Funding acquisition, Formal analysis, Data curation, Conceptualization. **Carlos E. Rodríguez-Jiménez:** Writing – review & editing, Writing – original draft, Visualization, Validation, Supervision, Software, Resources, Project administration, Methodology, Investigation, Funding acquisition, Formal analysis, Data curation, Conceptualization.

## Declaration of competing interest

The authors declare that they have no known competing financial interests or personal relationships that could have appeared to influence the work reported in this paper.
